# Uncontrolled Donors with Controlled Reperfusion after Sixty Minutes of Asystole: A Novel Reliable Resource for Kidney Transplantation

**DOI:** 10.1371/journal.pone.0064209

**Published:** 2013-05-30

**Authors:** Oleg N. Reznik, Andrei E. Skvortsov, Alexander O. Reznik, Alexey N. Ananyev, Alexey P. Tutin, Denis O. Kuzmin, Sergey F. Bagnenko

**Affiliations:** 1 Organ Transplant Department, Saint Petersburg Pavlov State Medical University, Saint Petersburg, Russia; 2 Organ Procurement Center, Saint Petersburg State Research Institute for Emergency, Saint Petersburg, Russia; University of Colorado School of Medicine, United States of America

## Abstract

**Background:**

Organ shortage leads to usage of kidneys from donors after sudden cardiac death, or uncontrolled donors (UDCD). Ischemic injury due to cessation of circulation remains a crucial problem that limits adoption of UDCD. Our clinical investigation was to determine the applicability of kidneys obtained from UDCD and resuscitated by extracorporeal perfusion *in situ* after 60 minutes of asystole.

**Methods:**

In 2009–2011, organ procurement service of St. Petersburg, obtained kidneys from 22 UDCD with critically expanded warm ischemic time (WIT). No patients were considered as potential organ donors initially. All donors died after sudden irreversible cardiac arrest. Mean WIT was 61.4±4.5 minutes. For kidney resuscitation, the subnormothermic extracorporeal abdominal perfusion with thrombolytics and leukocyte depletion was employed. Grafts were transplanted into 44 recipients. The outcomes of transplantation of resuscitated kidneys were compared to outcomes of 87 KTx from 74 brain death donors (BDDs).

**Results:**

Immediate functioning of kidney grafts was observed in 21 of the 44 recipients, with no cases of primary non function. By the end of the first post-transplant year there was an acute rejection rate of 9.1% (4 episodes of rejection) in the UDCD group versus 14.2% (13 episodes of rejection) in the BDD group. The actual 1-year graft survival rate was 95.5% (n = 42) in UDCD group, and 94.6% (n = 87) in BDD group. Creatinine levels at the end of the first year were 0.116±0.008 and 0.115±0.004 mmol/l in UDCD and BDD groups, respectively.

**Conclusions:**

UDCD kidneys with critically expanded WIT could be succefully used for transplantation if *in situ* organ “resuscitation” perfusion is included into procurement protocol. The results of 1-year follow-up meet the generally accepted criteria for graft survival and function. *In situ* reperfusion may exert a therapeutic effect on grafts before procurement. This approach could substantially expand the organ donors' pool.

## Introduction

The most pressing issue in organ transplantation is the critical shortage of donors. According to the United Network for Organ Sharing (UNOS), from January to March 2012 there were 114,951 patients awaiting transplantation and 3,375 donors, while only 6,838 operations were performed [1]. In Russia deceased organ donation is allowed only from BDDs and UDCDs, and according to the Registry of Russian Transplant Society, in 2011, an average of 3.3 organ donations per million of the entire Russian population took place [2]. To resolve the shortage of organs, a variety of approaches have been accepted recently [3]–[7]. Despite that, the transplant community cannot currently meet the demand for organs.

Alongside with organizational and educational efforts, widening the adoption of uncontrolled donation after cardiac death (UDCD) [8], or, according to Maastricht classification of 1993, the use of non-heart beating donors type II [9], may become a promising solution. Growing interest in the implementation of uncontrolled donation is covered in a number of recent papers [10]–[16]. However, serious ethical considerations [17], [18] and inevitable ischemic injury due to warm ischemic time (WIT) so far prevent wide acceptance of UDCD into practice. However, in the United States alone, the number of potential UDCDs was estimated at 22,000 per year [19].

For cases of controlled donation, successful use of extracorporeal perfusion technique with oxygenation as a bridge between the declaration of organ donor's death and the beginning of procurement procedure had been previously reported by several transplant teams [20], [21]. Indeed, without the development of special techniques, it would be difficult to resolve ethical and pathophysiological issues that hinder the routine usage of promising UDCD transplants supply. We hypothesize that the main component of ischemia-reperfusion damage to the procured organ is due to the «no-reflow syndrome» that is caused by blocking of capillaries by the clots of leuko- and thrombocytes and the swelling of endothelium.

Here, the subnormothermic extracorporeal perfusion *in situ* is considered as more than a supporting procedure [20], [21], but also as a resuscitation practice for kidneys from uncontrolled donors with critically prolonged WIT. We validate both the quality and the clinical applicability of resuscitated kidneys from uncontrolled deceased donors by reporting the nearest postoperative and 1-year follow-up outcomes.

## Methods

The design of this study, the protocols for perfusion, the organ procurement and the transplant procedures were approved by the Scientific Board and Ethics Committee of the Saint Petersburg State Research Institute for Emergency (Decision 7/0615/09) and authorized for clinical application by the Federal Advisory Service of The Ministry of Healthcare of the Russian Federation (Resolution N2010/299). All donation and perfusion procedures were approved by local Ethics Committees and Institutional Review Boards in each donor's hospitals of St. Petersburg.

In order to reveal the potential of donors who had had unexpected irreversible asystole, or cardiac death, the special perfusion procurement program was developed. In case of patient's sudden irreversible asystole within the hospital and after unsuccessful attempts of advanced cardiopulmonary resuscitation, the program is activated by the intensivists-on-duty (or “in-house” hospital transplant coordinator). After the confirmation and declaration of patient's death, the case is reported to the hospital administration. Next, the inform call to the St. Petersburg's Organ Procurement Centre (OPO) is performed. The distances between OPO and donor's hospitals in Saint Petersburg are from 6 to 16 miles. Usually, it takes the emergency vehicle with the procurement team and perfusion equipment on board 30 to 45 minutes to arrive after the call from ICU has been made. Before the arrival of the OPO-team at the hospital, the ICU specialists inject 25,000 IU of Heparin (Gedeon Richter, Germany) intravenously using central access. In order to disperse the anticoagulant within the donor's body, the ICU specialists are required to compress the donor's chest several times in a manner similar to the classic cardiopulmonary resuscitation procedure. According to the legal practice, these interventions with the exception of organ procurement do not require the consent from donors' relatives.

**Figure 1:**
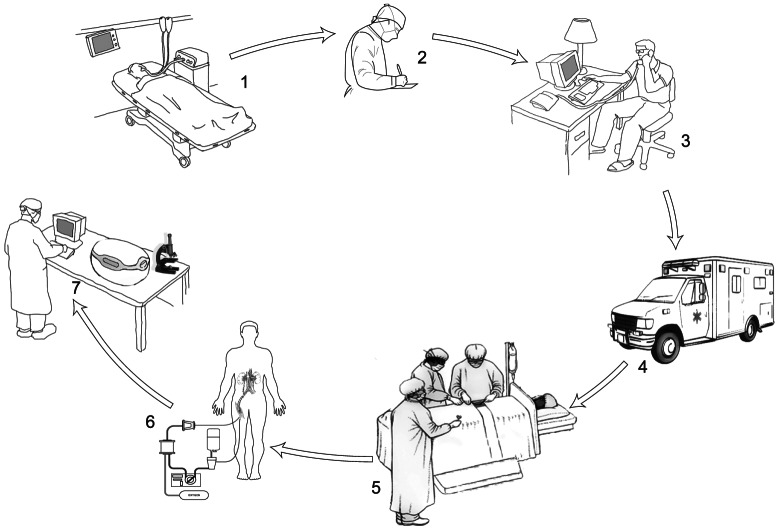
The logistics of the donor's procedures. 1: Death of patient after sudden irreversible cardiac arrest and the failure of resuscitation; 2: Hospital transplant coordinator fills out legal documents; 3: Activation of the program of donation by the call to regional transplant coordinator; 4: Arrival of the medical vehicle with perfusion and surgical team from the local OPO; 5: Cannulation of femoral vessels and catheterization of donor by procurement team; 6: Procedure of warm extracorporeal perfusion; 7: Possible addition to the described protocol – the verification of organ quality in OPO after the completion of in-hospital organ procurement procedure.

After that, the “no touch period” is maintained up to the arrival of the procurement team and the beginning of the *in situ* perfusion. The elapsed WIT extends from the declaration of patient death to the initiation of perfusion. No mechanical cardiac compression or continued ventilation is performed during WIT period. Fig. 1 shows the logistics of the procedure.

After the permission from the hospital administration is obtained, the donors are transferred to an operating room for catheterization of the femoral vessels and the *in situ* perfusion procedure is initiated. The consent and the legal authorization for inserting femoral catheters are not required.

Surgeon(s) perform the access to the femoral vessels on the right side of the body. The double balloon three-luminal catheters 16F (Balton, Warsaw, Poland) are inserted through the femoral artery for occlusion of the abdominal aorta at the level of bifurcation and just above the kidney arteries, thereby providing isolation of abdominal organs. The “outflow”-catheters are inserted into the inferior vena cava through the femoral vein and connected to the perfusion circuit, thereby establishing isolated abdominal perfusion. In order to prevent the collapse of the vena cava on the catheter, the hard-shell tubes are employed.

The perfusion circuits are set up simultaneously by the OPO team perfusionist. These circuits include the following components: leukocyte filter (LeukoGuard-6, Pall GmbH, Dreieich, Germany); perfusion module developed in the State Scientific Center of Russia/Central R&D Institute for Robotics and Technical Cybernetics (St. Petersburg, Russia); portable source of oxygen with system reducing gear (Alternative Science, St. Petersburg, Russia); hollow fibre oxygenator and 4-L hard-shell venous reservoir (Gish Vision Biomedical, Rancho Santa Margarita, CA, USA); and extracorporeal perfusion system tubes (Tianjin Plastics Research Institute, Tianjin, China).

**Figure 2:**
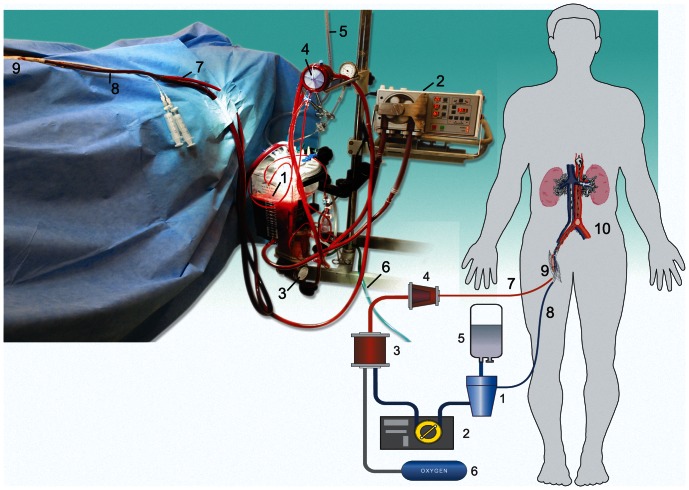
Schematics of the perfusion procedure. The components of the perfusion circuit include: 1: venous reservoir; 2: mechanical perfusion module; 3: oxygenator; 4: leukocyte filter; 5: preservation solution bag; 6: source of oxygen; 7: arterial line of the perfusion circuit; 8: venous line; 9: surgical access to femoral vessels; 10: zone of isolated abdominal perfusion.

Fig. 2 gives a general outline of the perfusion circuit. Perfusion circuit is assembled in 10–15 minutes. When venous and arterial ports have been connected to the perfusion circuit, *in situ* extracorporeal perfusion of an isolated abdominal region with membrane oxygenation and leukocyte depletion (LD) starts. For priming the circuit, we used up to 2L of Custodiol™ (HTK, histidine-tryptophan-ketoglutarate solution, Dr. F. Kohler Chemie GmbH, Bensheim, Germany). The relatively large volume of the priming solution is a technical requirement for filling hard-shell venous reservoirs and is needed to dilute donor's blood.

After the initiation of the perfusion procedure, in order to prevent leakage of the perfusate from the inferior vena cava to the venous pool of the lower left extremity, the left femoral vein are cross-clamped through a separate surgical access. The femoral accesses are thoroughly sewn and the donor's bodies are draped by surgical bed sheet.

Organ resuscitation procedure or, in other words, *controlled organ reperfusion procedure* consists of the following obligatory sub-procedures:

1. Abdominal *in situ* thrombolysis and heparinization through perfusion circuit
2. Elimination of leuko- and thrombocyte clots from the vascular bed of abdominal organs using the hemodilution and leukofilter incorporated into perfusion circuit
3. Subnormothermic extracorporeal membrane oxygenation of the perfusate.

In this procedure, the perfusate was, in fact, the modified donor blood. The following agents were sequentially injected into the perfusion circuit: 1.5 million units of Streptokinase (Belmedpreparat AO, Minsk, Belarus), 25,000 IU of Heparin (Gedeon Richter, Hamburg, Germany). During the first 30 minutes of perfusion, the perfusate flow was gradually increased from 500 ml/min to 3500 ml/min. The oxygen supply was maintained at 150–350 ml/min which corresponds to an average pO_2_ of 300.1±9.38 mm Hg. All procedures were performed under mild normothermic or subnormothermic conditions (27–32°C). Blood samples for leukocyte count and BGA were collected (Table 1). Table 1 shows the characteristics of perfusion procedures.

**Table 1:** Clinical parameters of the perfusion procedure.

Variable	(n = 22)
Hemoglobin, g/L	34.5±3.03
Hematocrit, %	0.32±0.02
pH of perfusate	7.32±0,03
Perfusion flow, initial, ml/min	500
Perfusion flow, final, ml/min	3500
Initial oxygen supply, ml/min	150
Final oxygen supply, ml/min	350
Average pO_2_ * in perfusate, mmHg	300.1±9.38
Average pCO_2_ ** in perfusate, mmHg	98.2±5.73
Duration of SNECP and LD***, min	145.5±6.1
Leukocyte count in perfusate, initial, ×10^9^/L	16.5±0.98
Leukocyte count in perfusate, final, ×10^9^/L	0.79±0.1

* pO_2_: partial pressure of oxygen.

** pCO_2_: partial pressure of carbon dioxide.

*** SNECP: subnormothermic extracorporeal perfusion; LD: leukocyte depletion.

The decrease of leukocyte count in the perfusion circuit was used as an indirect indication to start the surgical recovery procedure. A count of 1×10^9^ or lower was empirically considered as a satisfactory perfusion outcome (Table 1). On average, elimination of leukocytes from the abdominal perfusion circuit required no more than 120 minutes, the time that is sufficient to complete legal paperwork and obtain the next-of-kin consent. For initiation of surgical procurement procedure the permission of forensic pathologist is required. Although the perfusion procedures were initiated prior to the arrival of the forensic pathologist, the organ procurement procedures can be started only after they have made their decision and legal documentation has been filled out. The consent of next-of-kin was obtained in all cases. There were no organ donation refusals by relatives.

When legal and logistic procedures were completed, the final clinical decision to start a surgical procurement procedure was made. Laparotomy and kidney mobilization were performed and organ recovery commenced while the donor was still on the continuous extracorporeal perfusion. The perfusion procedure was terminated just before the surgical kidney explantation. Each kidney graft was placed in a separate plastic bag for subsequent static cold preservation in HTK solution.

Between 2009 and 2011, the donor procurement service was dispatched to attend 24 cases of unexpected death from irreversible asystole. In two cases, the attempts of extracorporeal resuscitation of kidneys failed due to technical problems, and the morphological material was taken for investigation. Ten donors died at the same hospital where the OPO was located, while other 14 donors died in 5 different emergency hospitals affiliated with the city procurement team under the regulations of the Government of Saint-Petersburg. All donors under 45 years either had sustained a head injury, had been suffering from an illness, or showed signs of impending brain death (Table 2). None of the donors displayed unstable hemodynamics, and, therefore, initially they were not considered as potential candidates for organ procurement. The cause of death of all the donors was sudden irreversible cardiac arrest following unsuccessful attempts of advanced CPR in ICU.

**Table 2:** Demographics and clinical characteristics of the donors after cardiac death.

Characteristic	UDCD (N = 22)	BDD (N = 74)	p
Age, years	41.8±2.1	45.18±1.2	>0.05
Gender:			-
Male	16 (72.7%)	38(51.4%)	
Female	6 (27.3%)	36(48.6%)	
Cause of death:			-
Brain injury	14 (63.6%)	17(22.9%)	
Cerebrovascular disease	8 (36.4%)	57(77.1%)	
Dopamine dose, µg/kg/min.	6.32±0.57	3.99±0.18	<0.05
Creatinine, before cardiac arrest, mmol/L,	0.079±0.005	0.072±0.002	>0.05
Diuresis during the last hour, L	0.46±0.09	0.6±0.04	<0.05
Warm ischemia, min	61.4±4.5	0	-

Mean WIT was 61.4±4.5 minutes (min = 20, max = 92), while mean extracorporeal subnormothermic perfusion time was 145.5±6.1 minutes (min = 105, max = 210). Despite the significant primary WIT, the colour and consistency of the abdominal organs during the procurement surgery were compatible with the typical characteristics of the vital organs comparable with brain-death donors. In all the 22 cases, the peristalsis of the intestine and ureters was observed. Moreover, in 14 cases, after one hour of the WIT, a spontaneous diuresis recovery (up to 100 ml) took place during the perfusion and explantation.

All kidneys from uncontrolled donors and from brain death donors were biopsied at the end of procurement procedure. Conventional needle biopsies were taken by a standard technique, stained with hematoxylin-eosin and Shiff's techniques, and then examined using light microscopy by a clinical pathologist.

Resuscitated kidneys were transplanted to 44 patients who had provided their informed consent to accept kidneys obtained from uncontrolled donors in accordance with the above-described preservation technique. Two forms of informed consent were filled in: the first one, concerning their agreement to be a donor's organ recipient, which is common to all patients on the kidney waiting list for transplantation, and the second one, informing about the study being conducted and possible complications, which are: delayed graft function and higher rejection rate.

To demonstrate the clinical applicability of kidneys from UDCD group, the transplantation results were compared to those of the transplantation of kidneys (n = 92) collected from 74 brain death donors (BDDs) during the same period of 2009–2010. Table 3 summarizes the data.

**Table 3:** Recipient data of early post-surgery and 1-year outcomes.

Characteristic	from UDCD Value (%) ± SD (N = 44)	from BDD Value (%) ± SD (N = 92)	p
Age, years	49.3±1.4	42.1±1.2	<0.05
Gender:			-
Male	24(54.5%)	62(67.4%)	
Female	20(45.5%)	30(32.6%)	
Type of dialysis:			-
Hemodialysis	37(84.1%)	76(82.6%)	
Peritoneal dialysis	7(15.9%)	16(17.4%)	
Years on dialysis prior to Tx	3.78±0.45	3.63±0.43	>0.05
Cause of end-stage renal disease:			-
Glomerulonephritis	40(90.9%)	66(71.7%)	
Pyelonephritis	1(2.3%)	15(16.3%)	
Polycystic kidney	3(6.8%)	11(11.9%)	
Current renal status:			-
Functioning graft	42(95.5%)	87(94.6%)	
Dialysis	2(4.5%)	3(3.3%)	
Deceased	-	2(2.2%)	
Cold ischemic time before transplantation, hours	13.9±0.64	14.2±0.6	>0.05
Graft function:			-
IGF^1^	21(47.7%)	58(63.1%)	
DGF^2^	23(52.3%)	34(36.9%)	
Number of dialysis/1 month; (for DGF group)	8.3±1.53	2.08±0.33	<0.05
Creatinine at 90 days, mmol/L	0.122±0.008	0.113±0.004	>0.05
Creatinine at 1 year, mmol/L	0.116±0.004	0.115±0.004^4^	>0.05
eGFR^3^, ml/min/1.73 m^2^	75.8±3.6	73.3±2.9	>0.05
Early acute rejection of kidney, first 3 months	2(4.6%)	9(9.8%)	-
Late acute rejection of kidney, 12 month	2(4.5%)	4(4.4%)	-
Surgical complications	1(2.5%)	2(2.2%)	-

1 IGF: immediate graft function.

2 DGF: delayed graft function.

3 eGFR: estimated glomerular filtration rate GFR Cockcroft = ((140−age) * mass (kg) [*0.85 if female])/72 * serum creatinine (mg/dl).

4 only 82 recipients have 1 years results.

In both groups, all the recipients underwent induction therapy by Baziliximab® 20 mg (Simulect, Novartis Pharma, Basel, Switzerland) on the first and 5^th^ day after transplant surgery and 3-component immunosuppressive therapy. Initial dose of Prograf® (Tacrolimus, Astellas Ltd, Astellas Ireland Co.) was 0.10–0.15 mg/kg/day; during the first month post-transplant. Target blood concentration of Tacrolimus was 9-7 ng/ml, and was lowered to 6-5 ng/ml by the third month. Initial dosage of CellCept® (Hoffman La Roche, Switzerland) was 2000 mg/day, and by the 21^st^ day the dosage of CellCept was lowered to 1000 mg/day. Initial dosage of Methypred (Methylprednisolon, Orion Corporation Pharma, Finland) was 0.3 mg/kg/day by the end of the first year the dosage was lowered to 0.05 mg/kg/day.

The criteria for kidney allograft rejection are as follows: the reduction of diuresis, the elevation of serum creatinine level, the increase of the transverse size of allograft and high RI (>1.0) evaluated by ultrasound study. In all episodes of rejection final diagnosis was based on a standard technique biopsy: needle biopsies were fixed in 10% formal saline, dehydrated and embedded in paraffin wax. Sections of 4 µm were cut and stained with hematoxylin-eosin for light microscopy by a clinical pathologist using Banff'05 classification criteria.

## Results

**Figure 3:**
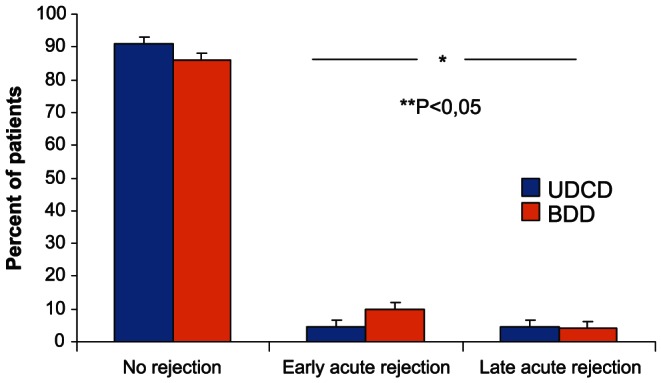
Early and late acute biopsy proven rejection. The occurence of early/late rejection episodes was higher in BDD group (*P*<0.05).

Table 1 shows the demographics and clinical characteristics of the donors. All donor characteristics were similar except the WIT. Table 3 shows the demographics and clinical characteristics of the recipients. In UDCD group, immediate function of kidney grafts were observed in 21 out of 44 cases (47,7%), while in BDD group immediate function were observed in 63.1% of cases (n = 58). There were no cases of primary non-function in UDCD group. Only 4 episodes of rejection (two early cases of rejection, within the first 3 months after surgery, and two late episodes of rejection, 6 month after transplantation) were observed at the end of the first year of observation (9.1%), while 13 episodes of rejection (9 early and 4 late) were noted in BDD group (14.2%) (Fig. 3).

**Figure 4:**
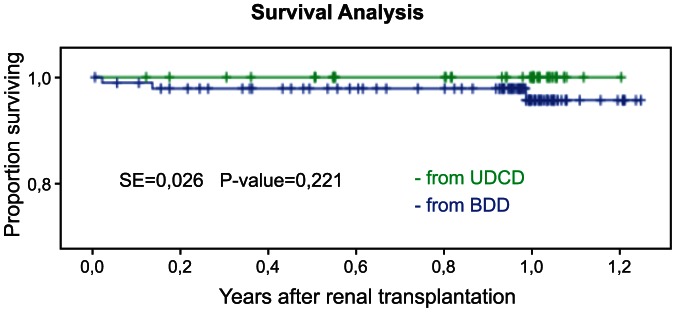
1-year Kaplan-Meier survival for kidney recipients showed similar patterns for BDD and UDCD groups (*P*<0.128, SE = 0.023).

**Figure 5:**
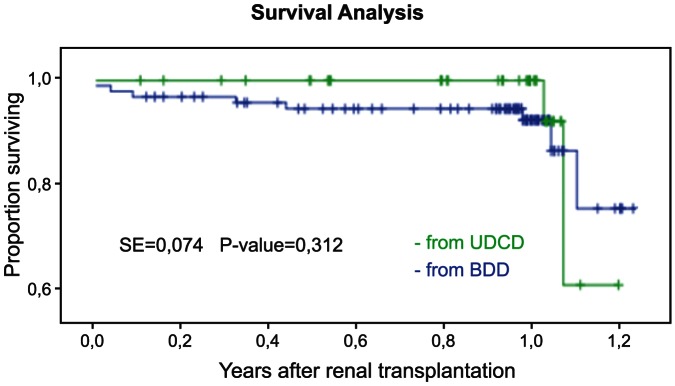
1-year Kaplan-Meier survival for kidney graphs showed similar patterns for BDD and UDCD groups (*P*<0.312, SE = 0.041).

The 1-year recipients survival in UDCD group was at 100%, while in BDD group the same survival parameter was 94.6% (n = 87) (Fig. 4). The 1-year graft survival in UDCD group was 95.5% (n = 42) (Fig. 5). The 1-year graft survival in BDD group was 94.6% (n = 87).

**Figure 6:**
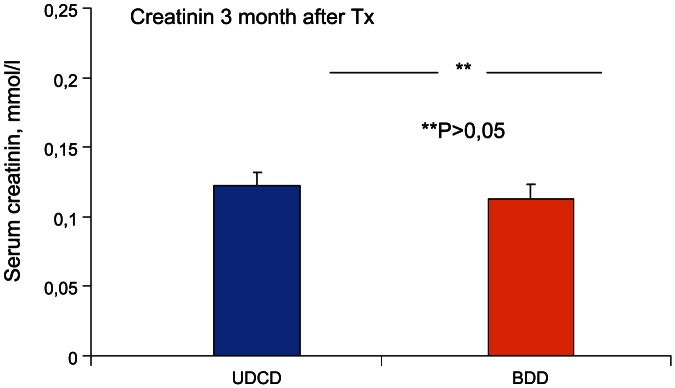
Comparison of serum creatinine levels in UDCD and BDD groups by the end of 3^rd^ month after transplantation (Tx). There were no significant differences between UDCD and BDD groups (*P*<0.262).

**Figure 7:**
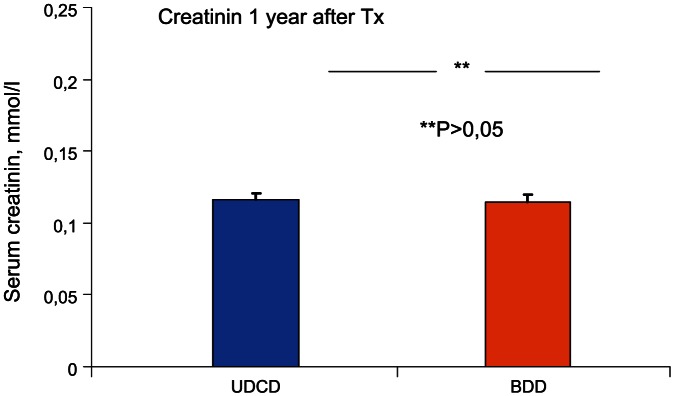
Comparison of serum creatinine levels in UDCD and BDD groups at the end of the first year after transplantation (Tx). There were no significant differences between UDCD and BDD groups (P<0.965).

By the end of the third month, the mean creatinine levels were 0.122±0.008 mmol/l and 0.113±0.004 mmol/L in UDCD and BDD groups, respectively (Fig. 6). By the end of the first year of observation, mean creatinine levels were 0.116±0.004 mmol/l and 0.115±0.004 in UDCD and BDD groups, respectively (Fig. 7).

**Figure 8:**
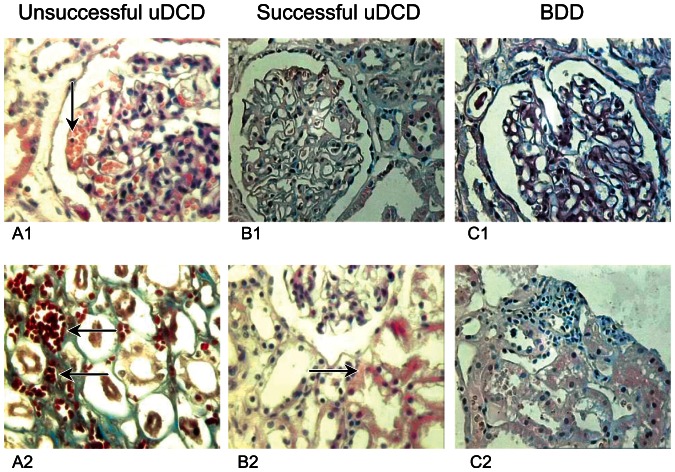
“Zero”-biopsy photomicrographs of specimen from the kidney grafts obtained at the end of the procurement procedure. Hematoxylin – Eosin and Shiff's staining. A1, A2 – the sample taken from an unsuccessful UDCD; B1,B2 – kidney biopsy performed in BDD; C1,C2 – the result of morphological investigation of specimen obtained from a successful UDCD. There are comments in the main text.

To prevent the thrombosis of the microcirculatory bed, we used thrombolytics (Streptokinase and Heparin) in all the cases. Total thrombosis of the glomeruli and tubuli was observed in the samples taken during unsuccessful UDCD procedures (A1, A2). In biopsy specimen from successful UDCD transplants taken at the end of procurement procedure (C1, C2), the mild interstitial edema was detected with only solitary red cells in the lumen of the microvessels of kidney, while there were no leucocytes. On the contrary, during the morphological investigation of samples obtained from BDD group (B1, B2), both the moderate edema and dilation of the tubules were observed; leucocyte infiltration of interstitial tissue and solitary thrombi in the lumen of the vessels (Fig. 8). Controlled reperfusion with leukocyte depletion and thrombolytics reduces leucocyte recruiting to kidney tissue and eliminates the blood clots which emerge during the preagonal condition followed by hemodynamic instability of the donors.

As evident from our study, the UDCD kidney transplantation outcomes are comparable to those involving the kidneys from “classical” brain death donor pool. The satisfactory outcomes of 1-year follow-up of our “resuscitated” kidney meet the generally accepted criteria for graft survival and function.

## Discussion

Excellent outcomes of the uncontrolled donation programs were previously demonstrated by several Spanish transplant programs that included the donors of Maastricht Classification Category I and II. Although all these programs routinely use extracorporeal support of abdominal organs with oxygenation, in previously described cases, the perfusion was started only after the emergency service had delivered an uncontrolled donor with sudden irreversible cardiac arrest to a hospital, literally “from the street”. In these cases, the WIT that was acceptable was a 15-minute gap between the cardiac arrest and the start of mechanical heart massage that preceded the perfusion [22]–[24]. There are other forms of extracorporeal support applicable to the deceased donations [20], [21], [25]. For example, Magliocca et al. (2005) reported good results of kidney transplantation with the perfusion support starting before or just immediately after cardiac death of donors [21]. In 2007, Fondevilla et al. reported excellent outcomes of the first clinical liver transplantation from uncontrolled donors, and later emphasized the use of the normothermic perfusion as a new approach to uncontrolled donation [13], [14], [26], [27].

However, these achievements do not resolve the main problem regarding uncontrolled donation after the cardiac death: to extend the maximum amount of time that may elapse between the declaration of death and the initiation of perfusion procedure with extracorporeal oxygenation, or, in other words, the maximum acceptable WIT. In 2010, Rojas-Pena et al. reported a 30-minute period to be the maximum acceptable WIT for kidney procurement, although in this case, the determination of WIT period may have reflected the characteristics of the animal model employed [28]. Interestingly, Moers et al. showed no beneficial effects of normothermic recirculation of ischemic kidneys in a small animal model [29]. One thing to explain the lack of perfusion benefits was the absence of thrombolytic pre-treatment and leukocyte depletion modules in the perfusion protocol.

Our clinical study is based on a theory that the underlying reason behind the ischemia-reperfusion graft injury is total microcirculation blockage by leukocyte and thrombocyte clots, following generalized endothelial swelling. These pathologic processes combined are the basis of the “no-reflow syndrome”.

In our clinical approach, we attempted to restore regional oxygen supply to the kidney after both pharmacological and mechanical, perfusion-assisted elimination of clots from the microvascular bed. This procedure rehabilitates ischemically damaged organs, possibly through the recovery of adenosine triphosphate (ATP) depot previously described by some authors [30]–[33]. In turn, this may restore the functioning of K^+^-Na^+^ cell membrane pump and reduce the endothelial edema. This study does not attempt to test this hypothesis scientifically, but rather provides empirical evidence in its favour. In clinical practice, evidence of the sub- and normothermic perfusion resuscitation effects are abundant. The first example of such kind was given in the pioneering works of Steen et al. [34]. These authors were primarily focused on leukocyte filtration during the perfusion recirculation procedure and have described the *ex vivo* reconditioning and repair of ischemically damaged human donor lungs [34]–[36]. Cypel et al. included the elimination of leukocytes as an obligatory step for *ex vivo* evaluation and resuscitation of donor lungs [37], [38]. Nevertheless, the understanding of the damage that leukocytes produce in the ischemia reperfusion process is limited; the only systematic study of isolated kidney hemoperfusion with thrombocytes and leukocyte depletion was reported by Nicolson et al. [39]. It is possible that damage by reperfusion could be successfully reduced if a leukofilter is employed, of a kind commonly used for cardiosurgical perfusion procedures. Then again, it should be noted that some of the studies had not done justice to the benefits of leukofiltration [40]. A stronger background to support the positive role of leukodepletion in reducing ischemia-reperfusion injuries could enhance our report. So would a more profound explanation of key mechanisms behind the ability of kidney tissue to maintain its vitality for up to one hour after the cessation of blood circulation.Many aspects of the mechanics of this protocol need further investigation.

Nevertheless, the resolution of organ shortage may be achieved by the adoption of some interdisciplinary approaches that are common in cardiosurgical practice, including the thrombolytic therapy routinely provided by cardiac ICUs. One example of the successful translation of this technology into practice of transplantation is the work of Talbot et al. that reported the inclusion of thrombolytic approach in organ donation practice in England [41]–[43]. Talbot's work resulted in 38 successful transplantations of kidneys from 19 donors after cardiac death with a WIT of up to 20 minutes [41]–[43]. Another example of cardiosurgical technique that is ready for adoption is the use of warm (sub- and normothermic) blood cardioplegia that provides much better myocardial protection as compared to the traditional cold crystalloid cardioplegia. In open heart surgery practice, the use of “warm” (25–27°C) heart blood recirculation instead of “cold” (12–14°C) cardiac arrest is known to reduce the mortality [44]–[46]. Accordingly, some experimental papers and reviews have reported that the use of sub- and normothermic blood perfusion in organ donation could improve the performance of ischemically damaged organs and, ultimately, lead to better transplantation outcomes [47]–[48].

For all UDCD programs, the most important ethical consideration is the respect of the donor autonomy [3]–[5]. Neither the medical nor the transplant team may act in an invasive manner to preserve organs before the final declaration of death, which will only be given after all possible resuscitation procedures have been provided for sudden irreversible cardiac arrest patient.

In order for the above described procurement technique to comply with ethical norms and legal regulations, two main prerequisites must be in place. First, one must firmly believe that they “resuscitate” the organs of a person who is certainly dead, i.e. after the declaration of death. Or, to be more specific, the manipulations (catheter insertion, the administration of heparin, short-term chest compression) must not accelerate the person's death, nor can those be started before all the means to save the person's life have been exhausted. In such a case donation-related manipulations remain “uncoupled” from intensive care measures: it is not the ICU staff, but the OPO team, which arrives at the hospital long after (at least 40 minutes) the patient dies, and the death has been registered, that performs femoral catheterization and the connection to perfusion circuit.

Another prerequisite is to start organ procurement only after the dead person's relatives have given their consent. The above described “resuscitation” perfusion and organ viability maintenance technique may provide a sustainable timeframe to inform the relatives and to ask for their consent. Minimally invasive procedures (femoral cannulation) in deceased persons do not violate the legislation of the Russian Federation. Should the relatives object to donation, the catheters will promptly be removed and the whole procedure will be terminated immediately. Therefore, restoring the viability of dead person's organs, even temporarily, with the hope to obtain the consent of the relatives for procurement, is ethically acceptable and does in no way contradict the Russian legislation.

Modern day standards of BDD organ procurement are affected by the shortage of these [40]. Various perfusion techniques to improve graft quality have been developed in look out for a wider donor pool [49], [50]. Yet, it was the post-procurement quality of kidney grafts that most of the previously implemented strategies aimed to improve. For baseline organ quality to become better, however, a wider choice is needed first, which is exactly a weak point of UDCD techniques, despite their strong potential. Implementing the extracorporeal perfusion technique that we have developed is, therefore, an organ salvation and maintenance technique, rather than a perfusion support procedure. Implementing the 'isolated abdominal controlled reperfusion', i.e. the “resuscitation” measures such as in situ use of thrombolytics, blood filtration and restoring the oxygenation of blood, compensates for or even eliminates the pathological consequences of uncontrolled asystole of as long as one hour, thereby opening up the availability of UDCD donor pool, previously considered beyond practical applications.

## Conclusions

Kidneys from uncontrolled donors with critically, up to one hour, expanded warm ischemic time could be succefully used for transplantation if *in situ* organ “resuscitation” perfusion is included into organ procurement protocol. The application of various types of controlled *in situ* reperfusion may exert a therapeutic effect on donor organs even before the procurement. The implementation of this approach can substantially expand the pool of available organs from uncontrolled donors.
